# *Acinetobacter baumannii* infections in Amazon Region driven by extensively drug resistant international clones, 2016-2018

**DOI:** 10.1590/0074-02760190232

**Published:** 2019-11-25

**Authors:** Raquel Vosges Caldart, Erica L Fonseca, Fernanda Freitas, Luiza Rocha, Ana Carolina Vicente

**Affiliations:** 1 Universidade Federal de Roraima, Boa Vista, RR, Brasil; 2 Fundação Oswaldo Cruz-Fiocruz, Instituto Oswaldo Cruz, Laboratório de Genética Molecular de Microrganismos, Rio de Janeiro, RJ, Brasil; 3 Laboratório Central de Saúde Pública de Roraima, Boa Vista, RR, Brasil

**Keywords:** international clone, extensively drug resistance, Acinetobacter baumannii, Amazon Region, *bla*_OXA-23_

## Abstract

**BACKGROUND:**

*Acinetobacter baumannii* is a leading cause of nosocomial infections. This species is characterised by the presence of pandemic lineages (International Clones) that present a broad antimicrobial resistance profile.

**OBJECTIVE:**

To perform the molecular epidemiology of carbapenem-resistant *A. baumannii* from a clinical setting in the Amazon Basin, and to characterise their antimicrobial resistance determinants.

**METHODS:**

The genetic relationship of carbapenem-resistant *A. baumannii* were assessed by pulsed-field gel electrophoresis (PFGE) and multilocus sequence typing (MLST). Class A, B and D β-lactamase genes were screened by polymerase chain reaction (PCR) and sequencing. The antimicrobial susceptibility profile was obtained by Disc-diffusion method and minimum inhibitory concentration (MIC) determination.

**FINDINGS:**

All carbapenem-resistant *A. baumannii* strains belonged to three international clones, IC-1, IC-5 and IC-6, the latter recently reported by the first time in Brazil. The major determinant of carbapenem resistance in IC-1 and IC-5 strains was *bla*_OXA-23_, associated with IS*Aba1* and IS*Aba3*, respectively, while IC-6 harboured the *bla*_OXA-72_.

**CONCLUSIONS:**

The *A. baumannii* epidemiology in Brazilian Amazon Region was unknown. It was demonstrated that *A. baumannii* XDR international clones were responsible for nosocomial infections in Boa Vista during 2016-2018, revealing that the epidemiological scenario of *A. baumannii* infections in Amazon Region resembles those from the cosmopolitan regions worldwide.

*Acinetobacter baumannii* has emerged in recent years as a leading cause of nosocomial infections associated with a longer hospital stay and higher mortality, representing a public health problem of major concern worldwide.^1^ *A. baumannii* presents the long-term ability to survive on inanimate surfaces, and this persistence seems to contribute to its person-to-person transmission, intra- and inter-hospital outbreak spread, and national and international clonal dissemination.^2^ Additionally, this species is characterised by remarkable capabilities for the acquisition of antibiotic resistance genes (ARGs).^3^ High-risk pandemic lineages, named international clones (ICs), presenting high capacity to persist in clinical environments and a broad antimicrobial resistance profile have been associated with outbreaks in several cosmopolitan regions around the world.^4^ ^,^ ^5^

Carbapenem resistance is a major therapeutic concern in *Acinetobacter* and it is usually mediated by carbapenem-hydrolysing class D β-lactamase (CHDL) from OXA Family, such as *bla*_OXA-23-like_, *bla*_OXA-58-like_, *bla*_OXA-24-like_, *bla*_OXA-143_ and *bla*_OXA-235_.^1^ ^,^ ^6^ ^,^ ^7^ In Brazil, the carbapenem resistance rates are around 80.7%^8^ as a consequence of *bla*_OXA-23_ dissemination by some pandemic lineages, such as those from CC15, CC79 and CC1 (international clone IC-1).^9^ ^,^ ^10^ ^,^ ^11^ The *bla*_OXA-23_ is frequently found downstream the IS*Aba1* sequence, which accounts for its mobilisation and supports its overexpression due to the presence of a strong promoter.^1^ ^,^ ^6^ ^,^ ^12^

The epidemiology of clinical *A. baumannii* and its antibiotic resistance determinants are concentrated in densely populated cosmopolitan cities from the South and Southeast Brazilian regions.^8^ ^,^ ^10^ ^,^ ^13^ Considering that Brazil is a country with continental dimensions and with contrasting demographical features, it is crucial to gain insights about the epidemiological scenario and the dynamics of carbapenem resistance in other geographical regions outside these cosmopolitan sites.

This study aimed to determine the molecular epidemiology of carbapenem-resistant *A. baumannii* strains from a clinical setting of the North region, and to characterise their carbapenem resistance determinants.

## MATERIALS AND METHODS

*Clinical data, bacterial strains and antimicrobial susceptibility test* - The General Hospital of Roraima (GHR), placed in Boa Vista, is a 281-bed tertiary healthcare medical unit, the largest in Roraima, which includes general wards, two intensive care units (ICUs) with 10 beds each, surgical centre and emergency.

From October, 2016 to May, 2018, 101 *A. baumannii* isolates were recovered from nosocomial infections cases of non-repetitive inpatients. From 101 isolates, 27 were resistant to carbapenem and these strains were characterised as described below (Table). Species identification was performed with the automated VITEK2, and confirmed by sequencing the 16S rRNA and the *bla*_OXA-51_ genes.

The antibiotic susceptibility profile was determined by disc-diffusion method according to clinical and laboratory standards institute (CLSI) guidelines,^14^ for the following antibiotics: gentamicin, amikacin, tobramycin, imipenem, meropenem, doripenem, ciprofloxacin, ampicillin/sulbactam, piperacillin/tazobactam, ticarcillin/clavulanic acid, cefotaxime, ceftazidime, cefepime, trimethoprim/sulphamethoxazole, tetracycline and minocycline. The minimum inhibitory concentration (MIC) of polymyxin B was assessed by the broth microdilution with antibiotic concentrations ranged from 0.1 μg/mL to 64 μg/mL. The current definition criteria for classifying *A. baumannii* antimicrobial resistance was applied.^15^ The carbapenemase production was assessed by the modified Hodge Test.^16^

**TABLE:** Epidemiological, phenotypic and genotypic features of the XDR *Acinetobacter baumannii* international clones found in General Hospital of Roraima, Boa Vista

Strains	Isolation date	PFGE	MLST (IC)	Ward	Clinical specimen	Carbapenemase activity	IS*Aba -bla*_OXA_
AB4332	Oct/16/16	B	ST78 (IC-6)	ICU	Tracheal secretion	+	OXA-72
AB4353	Oct/21/16	A	ST79 (IC-5)	ICU	Catheter tip	+	IS*Aba3*-OXA-23
AB5262	Dec/21/16	B	ST78 (IC-6)	ICU	Catheter tip	+	OXA-72
AB5375	Dec/29/16	B	ST78 (IC-6)	Traumatology	Tracheal secretion	+	OXA-72
AB49	Jan/03/17	C	ST1 (IC-1)	ICU	Catheter tip	+	IS*Aba1*-OXA-23
AB77	Jan/05/17	A	ST79 (IC-5)	others hospital wards	Wound secretion	+	IS*Aba3*-OXA-23
AB715	Feb/12/17	A	ST79 (IC-5)	Emergency	Bronchial aspirate	+	IS*Aba3*-OXA-23
AB1077	Mar/08/17	A	ST79 (IC-5)	ICU	Tracheal secretion	+	IS*Aba3*-OXA-23
AB1113	Mar/08/17	A	ST79 (IC-5)	ICU	Cerebrospinal fluid	+	IS*Aba3*-OXA-23
AB283	Apr/25/17	C	ST1 (IC-1)	ICU	Tracheal secretion	+	IS*Aba1*-OXA-23
AB08	Aug/31/17	B	ST78 (IC-6)	ICU	Blood	+	OXA-72
AB65	Sep/26/17	C	ST1 (IC-1)	others hospital wards	Bone tissue	+	IS*Aba1*-OXA-23
AB81	Oct/02/17	A1	ST79 (IC-5)	ICU	Tracheal secretion	+	IS*Aba3*-OXA-23
AB04-RR5	Jan/01/18	A1	ST79 (IC-5)	ICU	Blood	+	IS*Aba3*-OXA-23
AB07-RR5	Jan/14/18	C	ST1 (IC-1)	others hospital wards	Blood	+	IS*Aba1*-OXA-23
AB28-RR5	Jan/17/18	A	ST79 (IC-5)	ICU	Tracheal secretion	+	IS*Aba3*-OXA-23
AB37-RR5	Jan/19/18	A	ST79 (IC-5)	ICU	Tracheal secretion	+	IS*Aba3*-OXA-23
AB40-RR5	Jan/19/18	C	ST1 (IC-1)	ICU	Tracheal secretion	+	IS*Aba1*-OXA-23
AB39-RR5	Jan/21/18	C	ST1 (IC-1)	ICU	Tracheal secretion	+	IS*Aba1*-OXA-23
AB51-RR5	Jan/26/18	A	ST79 (IC-5)	ICU	Tracheal secretion	+	IS*Aba3*-OXA-23
AB07-RR6	Apr/23/18	C	ST1 (IC-1)	others hospital wards	Wound secretion	+	IS*Aba1*-OXA-23
AB04-RR6	Apr/25/18	A	ST79 (IC-5)	ICU	Catheter tip	+	IS*Aba3*-OXA-23
AB06-RR6	Apr/26/18	C	ST1 (IC-1)	ICU	Hepatic abscess	+	IS*Aba1*-OXA-23
AB01-RR6	Apr/29/18	B	ST78 (IC-6)	ICU	Catheter tip	+	OXA-72
AB05-RR6	Apr/29/18	A	ST79 (IC-5)	others hospital wards	Catheter tip	+	IS*Aba3*-OXA-23
AB02-RR6	May/03/18	C	ST1 (IC-1)	Emergency	Tracheal secretion	+	IS*Aba1*-OXA-23
AB03-RR6	May/15/18	B	ST78 (IC-6)	ICU	Tracheal secretion	+	OXA-72

ICU: intensive care unit; MLST (IC): multilocus sequence typing (international clone); PFGE: pulsed-field gel electrophoresis.

*Genotyping by pulsed-field gel electrophoresis (PFGE) and multilocus sequence typing (MLST)* - The clonal relationship among the carbapenem-resistant *A. baumannii* strains was establish by the *in situ* lysis technique, in agarose blocks as described previously and digested with 30U ApaI restriction enzyme. PFGE banding patterns were analysed and compared visually. Isolates were considered to be clonal when the macrorestriction DNA patterns differed by fewer than three bands.^17^

The MLST was performed using the Oxford an Pasteur schemes (https://pubmlst.org/abaumannii/) available in the *A. baumannii* MLST website (https://pubmlst.org/abaumannii/).^4^ ^,^ ^18^ Clonal complexes (CCs) were considered when sequence types (STs) shared five or more identical alleles taking into account the seven genes that are considered in the MLST schemes.^5^

*Detection of carbapenem resistance genes by polymerase chain reaction (PCR) and sequencing* - The presence of genes encoding class A (*bla*_KPC_, *bla*_GES_, *bla*_BKC_), class B (*bla*_IMP_, *bla*_VIM_, *bla*_GIM_, *bla*_SPM_, *bla*_SIM-1_, and *bla*_NDM-1_), and class D (*bla*_OXA-23-like_, *bla*_OXA-143-like_, *bla*_OXA-58-like_, *bla*_OXA-24/40-like_, and *bla*_OXA-51-like_) β-lactamases with carbapenemase activity was detected by PCR and sequencing in the carbapenem-resistant isolates as previously described.^19^ ^,^ ^20^ ^,^ ^21^ ^,^ ^22^ ^,^ ^23^ ^,^ ^24^

The presence of the IS*Aba1* upstream the *bla*_OXA_ genes was also investigated.^25^

## RESULTS AND DISCUSSION

The phenotypic analysis revealed that all carbapenem-resistant strains (n = 27) presented the XDR phenotype, since they were susceptible only to polymixin B, minocycline and tetracycline (Table). All strains were positive for the Modified Hodge Test, indicating carbapenemase production.

PFGE and MLST analyses demonstrated the concomitant occurrence of three XDR lineages in HGR from 2016 to 2018. The ST1^PAS^/ST109^OXF^ (Clone C; n = 9), ST79^PAS^/ST758^OXF^ (Clone A; n = 12) and ST78^PAS^/ST944^OXF^ (Clone B; n = 6), corresponded to the high-risk pandemic International Clone I (IC-1), International Clone V (IC-5) and International Clone VI (IC-6), respectively (Table).^20^ ^,^ ^26^ ^,^ ^27^ ^,^ ^28^ ^,^ ^29^ Previous studies had already reported the dissemination and the high prevalence of multidrug resistant *A. baumannii* from CC1 (ST1/IC-1), CC15 (ST180) and CC79 (ST79/IC-5) in Brazil.^9^ ^,^ ^10^ ^,^ ^11^ Here, it was demonstrated that CC1 and CC79 are also prevalent in a clinical setting from the Amazon Region (Table). Interestingly, considering Brazil, the IC-6 seems to be restricted, so far, to this clinical setting in the Amazon Region,^29^ although it has been involved with outbreaks worldwide since 2006.^5^ ^,^ ^30^ These findings stress the spatial temporal persistence and dissemination potential of these three pandemic lineages, since they also occurred in the Brazilian Amazon Region, at least, during 2016-2018.

The *bla*_OXA-23_, in association with IS*Aba* sequences, is one of the most widespread CHDL among *A. baumannii* in Brazil, and it has been disseminated in the country by the high-risk pandemic lineages from CC1 (IC-1), CC79 (IC-5) and CC15. However, most of these studies focused on clinical settings placed in the Southeast and South regions of Brazil.^2^ ^,^ ^9^ ^,^ ^10^ ^,^ ^11^ ^,^ ^26^ Similarly, we verified that *bla*_OXA-23_ was also the most prevalent carbapenemase gene among IC-1 and IC-5 XDR *A. baumannii* from Boa Vista (Table), and that it was found downstream IS*Aba1* and IS*Aba3*, explaining the observed carbapenem resistance in IC-1 and IC-5 strains, respectively. Instead, the IC-6 strains carried the carbapenemase *bla*_OXA-72_ gene flanked by XerC/XerD binding sites.^29^ However, in spite of that, it was previously demonstrated that, even in the absence of IS*Aba* sequences, *bla*_OXA-72_ had contributed to the carbapenem resistance.^29^ ^,^ ^31^

*In conclusion* - This study unraveled, by the first time, the epidemiological context of *A. baumannii* infections in a city from the Amazon Region. This scenario resembled that observed in cosmopolitan regions around the world, since it was verified that the nosocomial infections that occurred in Boa Vista from 2016 to 2018 was concomitantly caused by three XDR international clones, IC-1, IC-5 and IC-6, the latter only recently reported in Brazil (more precisely, in Boa Vista city). Such situation is probably due to the *A. baumannii* long-term ability to persist and survive in hospital environments, together with the person-to-person transmission and the global human mobility. Therefore, these findings provided a more complete picture concerning the importance of high-risk pandemic clones in the international dissemination of resistance, reinforcing the need of an epidemiological tracking of *A. baumannii* XDR strains and its associated carbapenemase coding genes even outside densely populated cosmopolitan regions.

## References

[1] Zarrilli R, Pournaras S, Giannouli M, Tsakris A (2013). Global evolution of multidrug-resistant Acinetobacter baumannii clonal lineages. Int J Antimicrob Agents.

[2] Rocha IV, Xavier DE, Almeida KRH, Oliveira SR, Leal NC (2018). Multidrug-resistant Acinetobacter baumannii clones persist on hospital inanimate surfaces. Braz J Infect Dis.

[3] Dijkshoorn L, Nemec A, Seifert H (2007). An increasing threat in hospitals multidrug-resistant Acinetobacter baumannii. Nat Rev Microbiol.

[4] Diancourt L, Passet V, Nemec A, Dijkshoorn L, Brisse S (2010). The population structure of Acinetobacter baumannii expanding multiresistant clones from an ancestral susceptible genetic pool. PLoS One.

[5] Karah N, Sundsfjord A, Towner K, Samuelsen Ø (2012). Insights into the global molecular epidemiology of carbapenem non-susceptible clones of Acinetobacter baumannii. Drug Resist Updat.

[6] Poirel L, Nordmann P (2006). Carbapenem resistance in Acinetobacter baumannii mechanisms and epidemiology. Clin Microbiol Infect.

[7] Neves FC, Clemente WT, Lincopan N, Paião ID, Neves PR, Romanelli RM (2016). Clinical and microbiological characteristics of OXA-23- and OXA-143-producing Acinetobacter baumannii in ICU patients at a teaching hospital, Brazil. Braz J Infect Dis.

[8] Rossi F, Girardello R, Cury AP, Di Gioia TSR, Almeida JN, Duarte AJS (2017). Emergence of colistin resistance in the largest university hospital complex of São Paulo, Brazil, over five years. Braz J Infect Dis.

[9] Camargo CH, Tiba MR, Saes MR, Vasconcellos FM, Santos LF, Romero EC (2016). Population structure analysis of carbapenem-resistant Acinetobacter baumannii clinical isolates from Brazil reveals predominance of clonal complexes 1, 15, and 79. Antimicrob Agents Chemother.

[10] Chagas TP, Carvalho KR, Santos ICO, Carvalho-Assef AP, Asensi MD (2014). Characterization of carbapenem-resistant Acinetobacter baumannii in Brazil (2008-2011) countrywide spread of OXA-23-producing clones (CC15 and CC79). Diagn Microbiol Infect Dis.

[11] Pagano M, Nunes LS, Niada M, Barth AL, Martins AF (2019). Comparative analysis of carbapenem-resistant Acinetobacter baumannii sequence types in southern Brazil from the first outbreak (2007-2008) to the endemic period (2013-2014). Microb Drug Resist.

[12] Meshkat Z, Amini Y, Sadeghian H, Salimizand H (2019). ISAba1/blaOXA-23-like family is the predominant cause of carbapenem resistance in Acinetobacter baumannii and Acinetobacter nosocomialis in Iran. Infect Genet Evol.

[13] Grosso F, Carvalho KR, Quinteira S, Ramos A, Carvalho-Assef AP, Asensi MD (2011). OXA-23-producing Acinetobacter baumannii a new hotspot of diversity in Rio de Janeiro?. J Antimicrob Chemother.

[14] CLSI - Clinical and Laboratory Standards Institute (2017). Performance standards for antimicrobial susceptibility testing 22th informational supplement (M100-S27) 2017.

[15] Magiorakos AP, Srinivasan A, Carey RB, Carmeli Y, Falagas ME, Giske CG (2012). Multidrug-resistant, extensively drug-resistant and pandrug-resistant bacteria an international expert proposal for interim standard definitions for acquired resistance. Clin Microbiol Infect.

[16] Lee K, Chong Y, Shin HB, Kim YA, Yong D, Yum JH (2001). Modified Hodge and EDTA-disk synergy tests to screen metallo-beta-lactamase-producing strains of Pseudomonas and Acinetobacter species. Clin Microbiol Infect.

[17] Tenover FC, Arbeit RD, Goering RV, Mickelsen PA, Murray BE, Persing DH (1995). Interpreting chromosomal DNA restriction patterns produced by pulsed field gel electrophoresis criteria for bacterial strain typing. J Clin Microbiol.

[18] Bartual SG, Seifert H, Hippler C, Luzon MA, Wisplinghoff H, Rodríguez-Valera F (2005). Development of a multilocus sequence typing scheme for characterization of clinical isolates of Acinetobacter baumannii. J Clin Microbiol.

[19] Woodford N, Ellington MJ, Coelho JM, Turton JF, Ward E, Brown S (2006). Multiplex PCR for genes encoding prevalent OXA carbapenemases in Acinetobacter spp. Int J Antimicrob Agents.

[20] Higgins PG, Dammhayn C, Hackel M, Seifert H (2010). Global spread of carbapenem-resistant Acinetobacter baumannii. J Antimicrob Chemother.

[21] Higgins PG, Poirel L, Lehmann M, Nordmann P, Seifer H (2009). OXA-143, a novel carbapenem-hydrolyzing class D beta-lactamase in Acinetobacter baumannii. Antimicrob Agents Chemother.

[22] Dallenne C, Da Costa A, Decre D, Favier C, Arlet G (2010). Development of a set of multiplex PCR assays for the detection of genes encoding important beta-lactamases in Enterobacteriaceae. J Antimicrob Chemother.

[23] Poirel L, Revathi G, Bernabeu S, Nordmann P (2011). Detection of NDM-1-producing Klebsiella pneumoniae in Kenya. Antimicrob Agents Chemother.

[24] Nicoletti AG, Marcondes MFM, Martins WMBS, Almeida LGP, Nicolás MF, Vasconcelos ATR (2015). Characterization of BKC-1 class A carbapenemase from Klebsiella pneumoniae clinical isolates in Brazil. Antimicrob Agents Chemother.

[25] Corvec S, Poirel L, Naas T, Drugeon H, Nordmann P (2007). Genetics and expression of the carbapenem-hydrolyzing oxacillinase gene blaOXA-23 in Acinetobacter baumannii. Antimicrob Agents Chemother.

[26] Royer S, de Campos PA, Araújo BF, Ferreira ML, Gonçalves IR, Batistão DWDF (2018). Molecular characterization and clonal dynamics of nosocomial blaOXA-23 producing XDR Acinetobacter baumannii. PLoS One.

[27] Tavares LCB, de Vasconcellos FM, de Sousa WV, Rocchetti TT, Mondelli AL, Ferreira AM (2019). Emergence and persistence of high-risk clones among MDR and XDR A baumannii at a Brazilian teaching hospital. Front Microbiol.

[28] Levy-Blitchtein S, Roca I, Plasencia-Rebata S, Vicente-Taboada W, Velásquez-Pomar J, Muñoz L (2018). Emergence and spread of carbapenem-resistant Acinetobacter baumannii international clones II and III in Lima, Peru. Emerg Microbes Infect.

[29] Fonseca ÉL, Caldart RV, Freitas FS, Morgado SM, Rocha LT, Santos RCD (2019). Emergence of XDR international clone IC-6 Acinetobacter baumannii carrying blaOXA-72 and blaCTX-M-115 in the Brazilian Amazon Region. J Glob Antimicrob Resist.

[30] Giannouli M, Cuccurullo S, Crivaro V, Di Popolo A, Bernardo M, Tomasone F (2010). Molecular epidemiology of multidrug-resistant Acinetobacter baumannii in a tertiary care hospital in Naples, Italy, shows the emergence of a novel epidemic clone. J Clin Microbiol.

[31] Fernández-Cuenca F, Rodríguez-Martínez JM, Gómez-Sánchez MC, Alba PD (2012). Production of a plasmid-encoded OXA-72 ß-lactamase associated with resistance to carbapenems in a clinical isolate Acinetobacter junii. Int J Antimicrob Agents.

